# Analysis of the value of nutritional status indexes (ALB, Hb, GNRI) in prognostic assessment of elderly patients with chronic heart failure

**DOI:** 10.3389/fcvm.2026.1736628

**Published:** 2026-02-24

**Authors:** Meng Xue, Conghan Wang, Lei Wang, Jianxiang Gao, Yanling Zhangsun

**Affiliations:** 1 Department of Cardiology, Xi'an People’s Hospital (Xi'an Fourth Hospital), Xi’an, China; 2 Department of General Practice, Xi'an People’s Hospital (Xi'an Fourth Hospital), Xi’an, China

**Keywords:** chronic heart failure in the elderly, Cox regression analysis, geriatric nutritional risk index, nutritional status indexes, prognosis, ROC

## Abstract

**Objective:**

This study evaluated the prognostic value of nutritional status indexes [albumin (ALB), hemoglobin (Hb), Geriatric Nutritional Risk Index (GNRI)] in elderly chronic heart failure (CHF) patients.

**Methods:**

A total of 190 elderly CHF patients were categorized into good (*n* = 142) and poor (*n* = 48) prognosis groups based on 1-year outcomes (rehospitalization or all-cause death). Clinical data, including cardiac function [New York Heart Association (NYHA) class], inflammatory markers, and nutritional indexes (ALB, Hb, GNRI), were analyzed. Spearman correlation was used to assess the relationship between nutritional markers and NYHA class. Patients were stratified by median ALB, Hb, and GNRI levels to compare poor prognosis incidence. Kaplan–Meier survival and Cox regression analyses identified prognostic factors, while Receiver Operating Characteristic (ROC) curves evaluated predictive performance.

**Results:**

The poor prognosis group exhibited significantly lower ALB, Hb, and GNRI levels (*P* < 0.001). These markers declined with worsening NYHA class (*P* < 0.001) and correlated negatively with cardiac function. Low ALB, Hb, and GNRI groups had higher poor prognosis rates (*P* < 0.001), confirmed by Kaplan–Meier analysis. Cox regression identified left ventricular ejection fraction (LVEF), N-terminal pro-B-type natriuretic peptide (NT-proBNP), NYHA class, ALB, Hb, and GNRI as independent prognostic factors. ROC analysis showed ALB [area under the curve (AUC) = 0.845], Hb (AUC = 0.884), and GNRI (AUC = 0.896) as strong predictors with high sensitivity/specificity.

**Conclusion:**

Reduced ALB, Hb, and GNRI levels are associated with poor CHF prognosis in elderly patients. These nutritional indexes offer reliable predictive value for clinical prognosis assessment.

## Introduction

Chronic heart failure (CHF) is a common and complex clinical syndrome in the field of cardiovascular disease, characterized by a weakened pumping function of the heart, which is unable to meet the body's metabolic demands (1). With the increasing trend of global population aging, the incidence of CHF in the elderly population has been increasing year by year, and it has become one of the major causes affecting the quality of life of the elderly (2, 3). Despite the advances in medical technology in recent years, the morbidity, mortality, and rehospitalization rates of elderly CHF patients remain high, which not only imposes a heavy financial burden on patients’ families, but also exerts tremendous pressure on healthcare resources (4, 5).

Malnutrition is a state of energy or nutrient deficiency caused by inadequate intake or impaired utilization. Malnutrition is associated with decreased functional status, impaired muscle function, decreased bone mass, immune dysfunction, anemia, decreased cognitive function, poor wound healing, delayed recovery from surgery, higher rates of hospitalization and readmission, and mortality, resulting in significant harm and economic burden for both the individual older adult and the family (6, 7). Studies have shown that nutritional status is closely related to the prognosis of many diseases, especially for elderly patients with CHF, and malnutrition may be one of the important risk factors for poor outcomes (8–10). Malnutrition leads to loss of muscle, adipose tissue, and bone mass, which increases the risk of death and the probability of rehospitalization in patients (11, 12). Therefore, accurate assessment of the nutritional status of elderly patients with CHF is important for the development of individualized treatment plans and improvement of patient prognosis.

Currently, commonly used nutritional assessment indicators in clinical practice include serum albumin (ALB), hemoglobin (Hb), and Geriatric Nutritional Risk Index (GNRI) (13, 14). Among them, ALB, as an important marker of protein nutritional status, has been widely used in the prognostic assessment of many diseases (15, 16); Hb level is closely related to anemia, which is a common and important complication in patients with chronic heart failure (17, 18); GNRI, as a comprehensive evaluation of the nutritional status of the index, can quickly assess the nutritional status of patients without additional GNRI is of high practical value as a comprehensive indicator of nutritional status that can rapidly assess the nutritional risk of patients without additional tests (19, 20).

However, there is a lack of systematic research on the specific role of these nutritional status indicators in elderly patients with chronic heart failure. The aim of this study is to retrospectively analyze the clinical data of elderly patients with chronic heart failure, to investigate the effects of ALB, Hb and GNRI on the prognosis of the patients, to clarify their predictive value in the elderly CHF population, and to provide a scientific basis for clinical practice.

## Materials and methods

### Study design and participants

This was a single-center, retrospective observational study. Consecutive sampling was employed, whereby all elderly patients diagnosed with CHF who were admitted to Xi'an People's Hospital (Xi'an Fourth Hospital) between January 2022 and January 2024 were initially screened. A total of 228 patients were assessed for eligibility. After applying the inclusion and exclusion criteria, 190 elderly CHF patients were finally enrolled as study subjects. The patients were categorized into good prognosis group (*n* = 142) and poor prognosis group (*n* = 48) based on the presence or absence of re-admission for heart failure or all-cause mortality at 1 year of follow-up. This study was conducted in accordance with the Declaration of Helsinki and was reviewed and approved by the Ethics Committee of Xi'an People's Hospital (Xi'an Fourth Hospital). The requirement for individual informed consent was waived by the ethics committee for this retrospective analysis of anonymized clinical data.

### Inclusion criteria

(1) Met the diagnostic criteria for chronic heart failure (21); (2) Age ≥ 60 years old; (3) Complete clinical and pathological data and follow-up data.

### Exclusion criteria

Presence of malignant tumors in other parts of the body; (2) Severe damage to vital organs such as heart, brain, liver, and kidneys; (3) Transfusion of albumin or other blood products in the last week; (4) Hormone or immunosuppressant users in the last 3 months; (5) Incomplete clinical information.

### Collecting information

Clinical baseline data of all elderly CHF patients were collected and organized, including age, gender, smoking history, drinking history, hypertension, hyperlipidemia, diabetes, Cardiac ultrasound values left atrial diameter (LAD), left ventricular end-systolic diameter (LVDs), left ventricular end-diastolic diameter (LVDd), left ventricular ejection fraction (LVEF), serum high-sensitivity C-reactive protein (hs-CRP), N-terminal pro-B-type natriuretic peptide (NT-proBNP), and New York Heart Association (NYHA) cardiac function class, serum albumin (ALB), hemoglobin (Hb) levels, etc. Doppler echocardiography was performed to detect cardiac echocardiographic indices using a PHILIPS Affiniti 70W (IL, USA) to record LAD, LVDs, LVDd, and LVEF.

Fasting elbow venous blood (5 mL) was collected from all patients on the early morning of admission, placed in a vacuum blood collection tube without anticoagulant, and naturally agglutinated at room temperature for 30 min. After blood coagulation, the samples were centrifuged at 2,000 r/min for 20 min, and the upper serum was collected and stored at −80 °C for backup testing. The kits for serum ALB, hs-CRP, and NT-proBNP were purchased from Nanjing Jiancheng Bioengineering Institute (Nanjing, China). The Hb detection kit was provided by Tianjin Yueteng Biotechnology Co., Ltd. (Tianjin, China). All detections were performed strictly in accordance with the kit instructions, and the instruments used were calibrated regularly to ensure the reliability of test results.

According to the NYHA functional class, the cardiac function class of the enrolled patients was II-IV. In addition, the Geriatric Nutritional Risk Index (GNRI) was calculated according to the formula (22): GNRI = 1.489 × serum albumin (g/L) + 41.7 × actual weight (kg)/ideal weight. Ideal weight was calculated using the Lorenz formula: height (cm)−100−[height (cm)−150]/4) for men and height (cm)−100−[height (cm)−150]/2.5) for women.

### Follow-up and prognostic evaluation

Elderly patients with CHF were discharged from the hospital and followed up by outpatient clinic or telephone for 1 year and every 3 months. The follow-up time end point was set to January 2025, and the occurrence of another heart failure admission or all-cause death was defined as poor prognosis, and the incidence of poor prognosis in patients was counted.

### Statistical analysis

A *post-hoc* power analysis was performed using G*Power software (version 3.1). With a two-sided alpha of 0.05, effect size d = 0.5, and the actual sample sizes (good prognosis group: *n* = 142; poor prognosis group: *n* = 48), the achieved statistical power (1—β) was 0.85, which exceeds the conventional threshold of 0.80 for cohort studies, indicating adequate sample size for statistical inference regarding the primary outcome. GraphPad Prism 9.5.0 software (GraphPad Software Inc., San Diego, CA, USA) and SPSS 27.0 statistical software (SPSS, Inc, Chicago, IL, USA) were used for statistical analysis and graphing of data. The Shapiro–Wilk test was used to test for normal distribution; normally distributed measures were expressed as mea*n* ± standard deviation, independent samples *t*-tests were used between two groups, and one-way ANOVA analyses were used between multiple groups; non-normally distributed measures were expressed as median (interquartile range), and Mann–Whitney U test was used for comparison between two groups, and multiple groups Kruskal–Wallis test was used. Count data were expressed as number of cases and percentage using Chi-square test. Spearman correlation coefficient analysis was used to assess the correlation between the indicators. Kaplan–Meier test and log-rank test for comparison of distributions were used to plot the curve of poor prognosis in elderly CHF patients. Cox regression was used to analyze the factors influencing the prognosis of elderly CHF patients. ROC curves were used to assess the predictive value of nutritional status indexes (ALB, Hb, GNRI) on poor prognosis in elderly CHF patients. *P* values were obtained from two-sided tests, and *P* < 0.05 was considered a statistically significant difference.

## Results

### Clinical baseline characteristics of the enrolled population

In this study, 190 elderly patients with CHF were included as research subjects and were divided into a good prognosis group (*n* = 142) and a poor prognosis group (*n* = 48) according to the prognosis of the patients. Comparative analysis of the clinical baseline data of the two groups of patients was performed and analyzed, as shown in Table 1, there were no significant differences in terms of age, gender, smoking history, drinking history, hypertension, hyperlipidemia, diabetes, and LAD (all *P* > 0.05), but there were significant differences in LVDs, LVDd, LVEF, hs-CRP, NT-proBNP and NYHA cardiac function class (all *P* < 0.05).

**Table 1:** Clinical baseline information of the enrolled population.

Variable	Good prognosis group (*n* = 142)	Poor prognosis group (*n* = 48)	t/*χ*2/Z	*P*-value
Age (years)	72.82 ± 3.52	73.50 ± 3.56	1.147	0.253
Gender (*n*, %)				
Male	81 (57.04%)	29 (60.42%)	0.168	0.682
Female	61 (42.96%)	19 (39.58%)		
Smoking history (n, %)	58 (40.85%)	21 (43.75%)	0.125	0.724
Drinking history (n, %)	48 (33.80%)	18 (37.50%)	0.216	0.642
Hypertension (n, %)	63 (44.37%)	23 (47.92%)	0.183	0.669
Hyperlipidemia (n, %)	43 (30.28%)	17 (35.42%)	0.438	0.508
Diabetes (n, %)	18 (12.68%)	7 (14.58%)	0.114	0.735
Cardiac ultrasound values	–	–		
LAD (mm)	37.00 (36.00, 38.00)	37.00 (36.00, 38.00)	−0.237	0.815
LVDs (mm)	36.00 (36.00, 37.00)	38.00 (37.00, 39.75)	−6.328	<0.001
LVDd (mm)	51.00 (50.00, 51.00)	53.00 (52.00, 54.00)	−8.547	<0.001
LVEF (%)	49.42 ± 3.60	46.85 ± 3.21	4.372	<0.001
hs-CRP (mg/L)	5.83 ± 0.70	7.09 ± 1.08	9.293	<0.001
NT-proBNP (ng/L)	672.10 ± 25.88	861.00 ± 33.36	40.510	<0.001
NYHA Cardiac Function Classification	–	–		
II	86 (60.56%)	1 (2.08%)	103.620	<0.001
III	51 (35.92%)	14 (29.17%)		
IV	5 (3.52%)	33 (68.75%)		

LAD: left atrial diameter; LVDs: left ventricular end-systolic diameter; LVDd: left ventricular end-diastolic diameter; LVEF: left ventricular ejection fraction; hs-CRP: high-sensitivity C-reactive Protein; NT-proBNP: N-terminal pro-B-type natriuretic peptide; NYHA: New York Heart Association. Association. Counting data were expressed as the number of cases and percentage by Chi-square test. Measurement data conforming to the normal distribution were expressed as the mean ± standard deviation, and the comparison between two groups was performed by t-test; non-normally distributed measurements were expressed as the median (interquartile range), and the comparison between two groups was performed by the Mann–Whitney U test. *P* < 0.05 was considered as statistically significant difference. Data from this retrospective clinical analysis.

### Comparison of nutritional status indexes (ALB, Hb, GNRI) in two groups of elderly CHF patients

**Figure 1:**
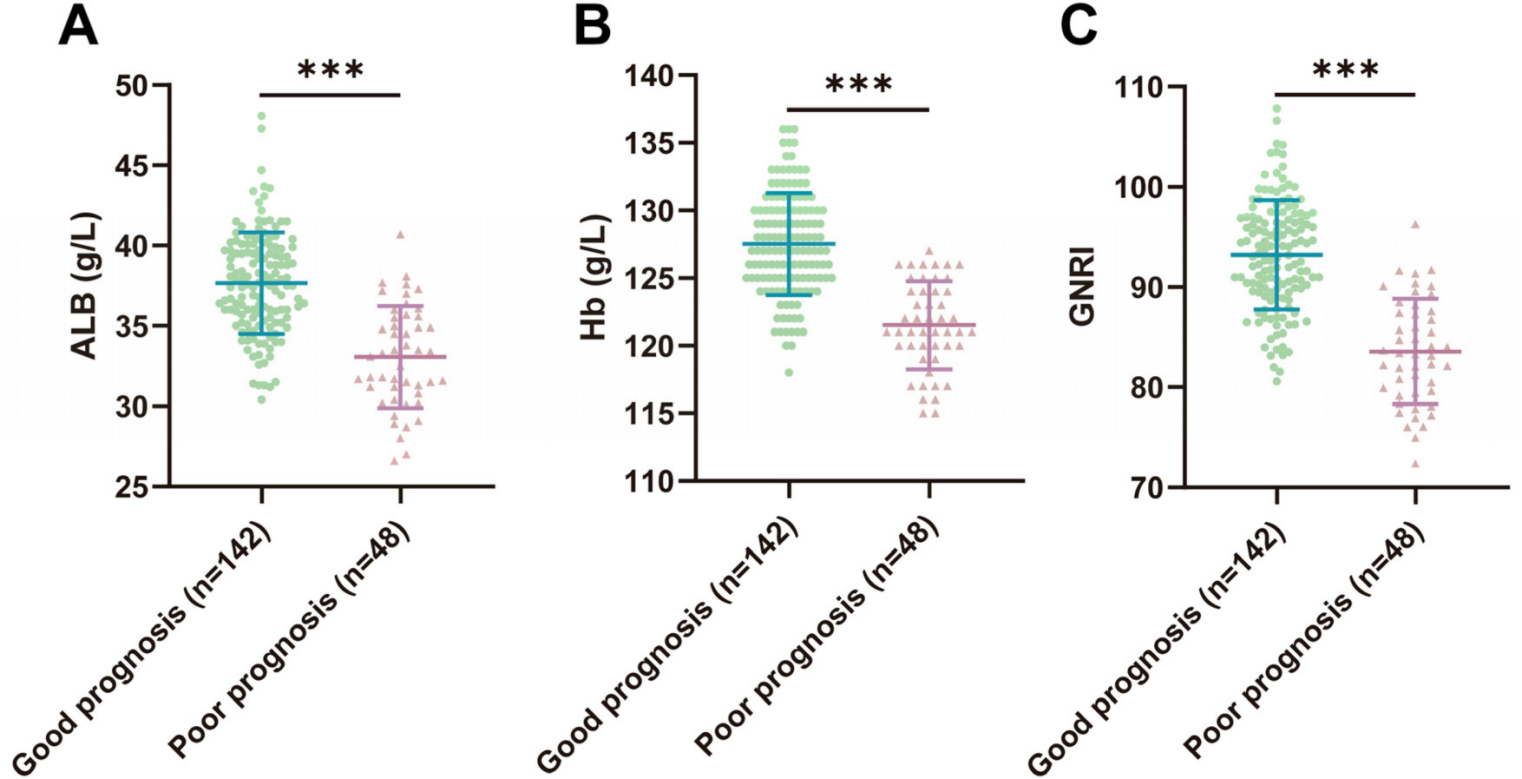
Comparison of nutritional status indexes (ALB, Hb, GNRI) between two groups of elderly CHF patients. **(A,B)** Laboratory examination of ALB and Hb levels; **(C)** Assessment of the nutritional status of the patients according to the GNRI formula, GNRI = 1.489 × serum albumin (g/L) + 41.7 × actual weight (kg)/ideal weight. The graphs conform to the normal distribution and are expressed as mean ± standard deviation using the independent samples *t*-test. *** indicates *P* < 0.001. Source: Data from this retrospective study. Figures were generated using GraphPad Prism 9.5.0.

We compared the nutritional status indexes (ALB, Hb, GNRI) of two groups of elderly CHF patients. The results showed that the levels of ALB, Hb, and GNRI were significantly decreased in elderly CHF patients in the poor prognosis group compared to patients in the good prognosis group (*P* < 0.001) (Figures 1A,B). The above results indicated that there was a significant reduction in nutritional status in elderly patients with poor prognosis of CHF.

### Different cardiac function classifications nutritional status indexes (ALB, Hb, GNRI) levels in elderly CHF patients

**Figure 2:**
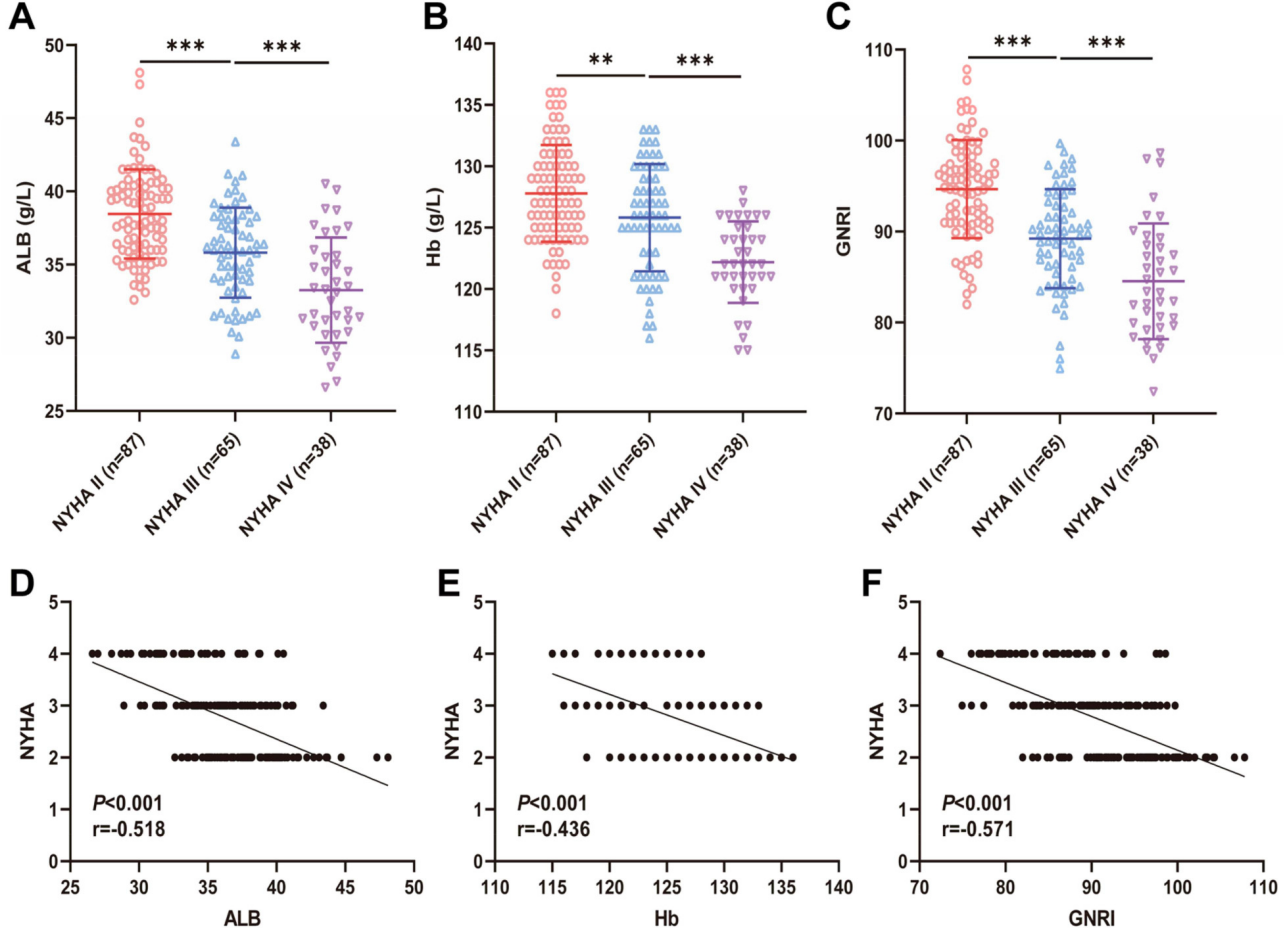
Levels of nutritional status indexes (ALB, Hb, GNRI) in elderly CHF patients with different cardiac function classifications. **(A,B)** Laboratory examination of serum ALB and Hb levels; **(C)** Nutritional status of the patients was evaluated according to the GNRI formula,GNRI = 1.489 × serum albumin (g/L) + 41.7 × actual weight (kg)/ideal weight; **(D-F)** Spearman's correlation coefficient to analyze the correlation between ALB, Hb, GNRI and NYHA cardiac function grading of the elderly CHF patients. r is the correlation coefficient. *r* is the correlation coefficient. Normal distribution was conformed in **(A–C)**, expressed as mean ± standard deviation, and analyzed by one-way ANOVA. ** indicates *P* < 0.01 and *** indicates *P* < 0.001. Source: Data from this retrospective study. Figures were generated using GraphPad Prism 9.5.0.

We compared the levels of nutritional status indexes (ALB, Hb, GNRI) in elderly CHF patients with different cardiac function classifications, and found that the levels of ALB, Hb, and GNRI gradually decreased in elderly CHF patients as NYHA cardiac function classifications increased (all *P* < 0.01) (Figures 2A–C). In addition, we further analyzed the correlation between the levels of nutritional status indexes (ALB, Hb, GNRI) and NYHA cardiac function grading in elderly CHF patients, and Spearman's correlation analysis showed that the levels of ALB, Hb, and GNRI were significantly negatively correlated with NYHA cardiac function grading in elderly CHF patients, respectively (*P* < 0.001, r = −0.518; *P* < 0.001, r = −0.436; *P* < 0.001, r = −0.571) (Figures 2D–F). These results suggest that the worse the cardiac function, the worse the nutritional status of elderly CHF patients.

### Low expression of ALB, Hb, and GNRI significantly increases the incidence of poor prognosis in elderly CHF patients

**Figure 3:**
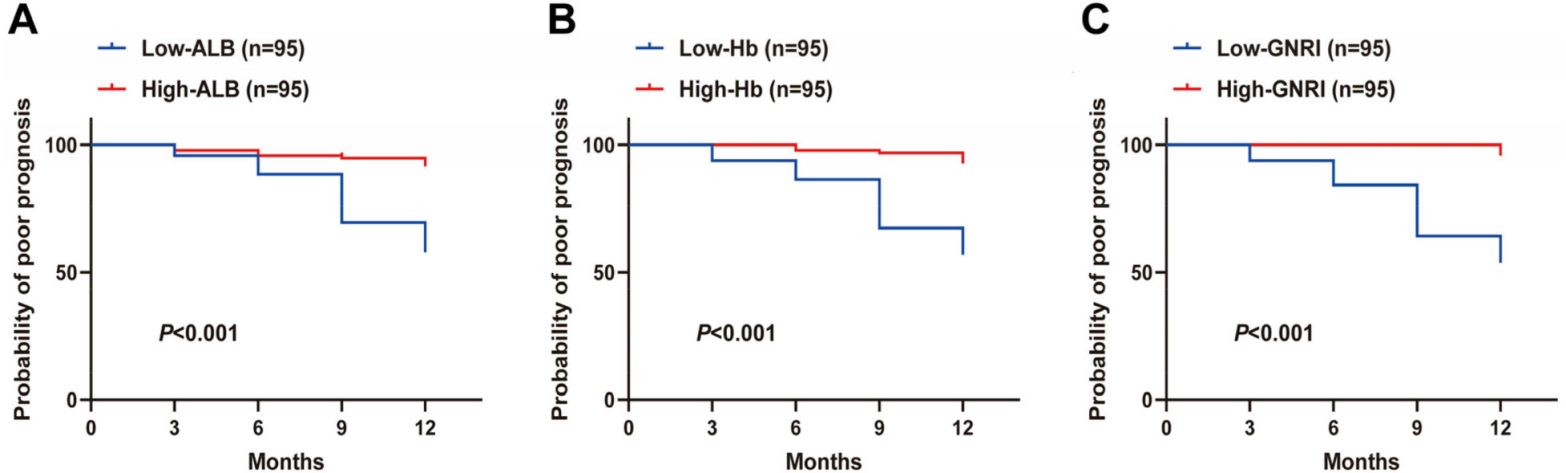
Poor prognostic curve of elderly CHF patients with different levels of nutritional status indexes (ALB, Hb, GNRI). **(A–C)** Log-rank analysis to plot poor prognostic curves in elderly CHF patients with ALB, Hb, and GNRI levels. Source: Data from this retrospective study. Figures were generated using GraphPad Prism 9.5.0.

In order to further investigate the relationship between nutritional status indexes (ALB, Hb, GNRI) and the prognosis of elderly CHF patients, the median values of ALB level (36.40), Hb level (126), and GNRI level (90.96) of elderly CHF patients were used as the cut-off values, and the elderly CHF patients were categorized into the ALB high-low-expression group, Hb high-low-expression group, and GNRI high-low-expression group, and compared the incidence of poor prognosis of elderly CHF patients in the two groups, and the results showed that the incidence of poor prognosis of patients in the low -ALB group, the low -Hb group, and the low -GNRI group was 83.33%, 85.42%, and 91.67%, respectively, which was significantly higher than that of the high -ALB group (16.67%), the high-Hb group (14.58%) and the high-GNRI group (8.5%) (all *P* < 0.001) (Table 2). Subsequently, poor prognostic Kaplan–Meier curves of elderly CHF patients were plotted based on Kaplan–Meier curves and log-rank test (log-rank) distribution comparison, and the results showed that the KM curve of patients with low expression of ALB, Hb and GNRI shifted to the left compared with those with high expression of ALB, Hb and GNRI. That is, the poor prognosis rate of elderly CHF patients with low expression of ALB, Hb and GNRI was significantly reduced (all *P* < 0.001) (Figures 3A,B). The above results indicate suggest that low expression of ALB, Hb, and GNRI significantly increases the risk of poor prognosis in elderly CHF patients.

**Table 2:** The relationship between the levels of ALB, Hb and GNRI and the prognosis of elderly patients with CHF.

Group	Good prognosis	Poor prognosis	χ2	*P*-value
Low-ALB (*n* = 95)	55 (38.73%)	40 (83.33%)	28.541	<0.001
High-ALB (*n* = 95)	87 (61.27%)	8 (16.67%)		
Low- Hb (*n* = 95)	54 (38.03%)	41 (85.42%)	32.221	<0.001
High-Hb (*n* = 95)	88 (61.97%)	7 (14.58%)		
Low- GNRI (*n* = 95)	51 (35.92%)	44 (91.67%)	44.601	<0.001
High-GNRI (*n* = 95)	91 (64.08%)	4 (8.33%)		

Count data were expressed using the number of cases and percentages, and differences were considered statistically significant using the Chi-square test *P* < 0.05. Data from this retrospective clinical analysis.

### Univariate and multivariate cox regression analysis of prognostic factors in elderly patients with CHF

Using the follow-up time as the time variable and the prognosis of elderly CHF patients as the dependent variable (good prognosis = 0, poor prognosis = 1), the pathological baseline characteristics of the population enrolled in Table 1 age, gender, smoking history, drinking history, hypertension, hyperlipidemia, diabetes, LAD, LVDs, LVDd, LVEF, hs-CRP, NT-proBNP, NYHA cardiac function Classification and ALB, Hb, and GNRI were taken as independent variables for univariate analysis, which showed that LVDs, LVDd, LVEF, hs-CRP, NT-proBNP, NYHA cardiac function classification, ALB, Hb, and GNRI were significantly correlated with the prognostic status of elderly patients with CHF (all *P* < 0.05, Table 3); Subsequently, the variables with statistically significant differences in the univariate Cox regression analysis were included in the multivariate Cox regression model. The results showed that the multivariate results indicated that LVEF, NT-proBNP, NYHA cardiac function classification, ALB, Hb, and GNRI were independent prognostic factors affecting elderly CHF patients (all *P* < 0.05, Table 3).

**Table 3:** Univariate and multivariate Cox regression analysis of prognostic factors in elderly patients with CHF.

Variable	Univariable		Multivariable	
	HR (95% CI)	*P*	HR (95% CI)	*P*
Age (years)	1.051 (0.971–1.138)	0.218	/	/
Gender (*n*, %)	1.194 (0.670–2.130)	0.548	/	/
Smoking history (*n*, %)	1.046 (0.592–1.851)	0.876	/	/
Drinking history (*n*, %)	1.085 (0.605–1.947)	0.784		
Hypertension (*n*, %)	1.104 (0.626–1.944)	0.733	/	/
Hyperlipidemia (*n*, %)	1.182 (0.654–2.136)	0.579	/	/
Diabetes (*n*, %)	1.121 (0.503–2.498)	0.780	/	/
LAD (mm)	1.050 (0.844–1.306)	0.663	/	/
LVDs (mm)	1.743 (1.483–2.050)	<0.001	1.007 (0.833–1.218)	0.944
LVDd (mm)	1.913 (1.648–2.220)	<0.001	1.043 (0.844–1.290)	0.695
LVEF (%)	0.858 (0.794–0.927)	<0.001	1.114 (1.003–1.238)	0.045
hs-CRP (mg/L)	2.710 (2.092–3.511)	<0.001	1.118 (0.829–1.508)	0.466
NT-proBNP (ng/L)	1.019 (1.015–1.023)	<0.001	1.013 (1.007–1.019)	<0.001
NYHA cardiac function classification	7.470 (4.496–12.411)	<0.001	2.220 (1.188–4.148)	0.012
ALB (g/L)	0.761 (0.704–0.823)	<0.001	1.279 (1.052–1.555)	0.014
Hb (g/L)	0.751 (0.699–0.807)	<0.001	0.872 (0.792–0.960)	0.005
GNRI	0.835 (0.799–0.873)	<0.001	0.853 (0.756–0.962)	0.010

Data from this retrospective clinical analysis.

### Analysis of the value of ALB, Hb, and GNRI in predicting poor prognosis in elderly patients with CHF

**Figure 4:**
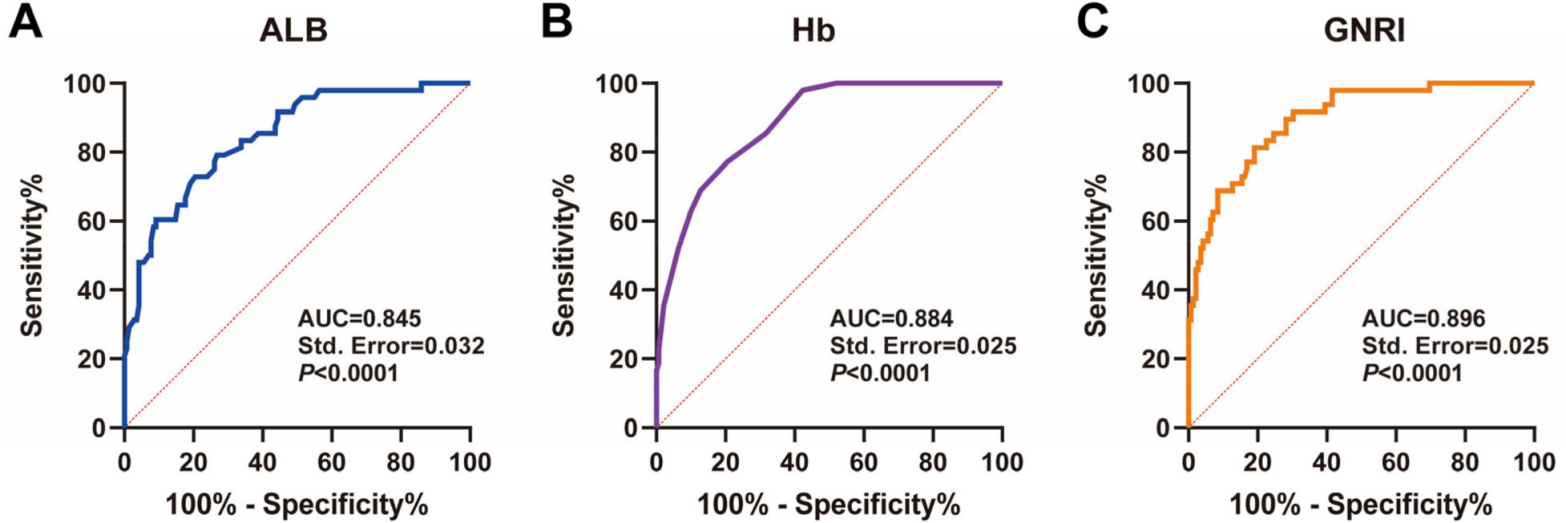
Analysis of the value of ALB, Hb, and GNRI in predicting poor prognosis in elderly patients with CHF. **(A–C)** ROC curve analysis of ALB, Hb, and GNRI in elderly patients with CHF. Source: Data from this retrospective study. Figures were generated using GraphPad Prism 9.5.0.

Based on these results, we used ROC curve analysis to further assess the predictive value of ALB, Hb, and GNRI for poor prognosis in elderly CHF patients. The results showed that the area under the ROC curve (AUC) of ALB for predicting poor prognosis in elderly CHF patients was 0.845, with a sensitivity of 72.92%, a specificity of 79.58%, and a cutoff value of 35.05; the AUC of Hb for predicting poor prognosis in elderly CHF patients was 0.884, with a sensitivity of 77.08%, a specificity of 79.58%, with a cutoff value of 124.50; GNRI predicted poor prognosis in elderly CHF patients with an AUC of 0.896, a sensitivity of 81.25%, specificity was 80.99% and cutoff value was 88.56 (Figures 4A–C). The above results suggest that nutritional status indexes (ALB, Hb, GNRI) has a good predictive value for poor prognosis in elderly CHF patients.

## Discussion

Chronic heart failure is a group of syndromes occurring in the decompensated phase of end-stage heart disease, and is prevalent in the elderly. As the main group of chronic heart failure, the elderly have nutritional and metabolic disturbances — such as multimorbidity, multiple organ disease, diarrhea, and bloating, which can trigger acute exacerbation of CHF, worsen the clinical course, and increase the morbidity, mortality, and readmission rates (23, 24). Malnutrition is defined as insufficient, excessive or imbalanced intake of energy and/or nutrients. As malnutrition worsens in elderly patients with CHF, the reduction in muscle mass and loss of muscle function can further lead to cardiac cachexia, which enters into a vicious cycle of “malnutrition-cachexia”, aggravating fluid retention and inflammation, ultimately leading to a poor prognosis (25, 26). Timely and effective nutritional risk assessment and supportive interventions can improve the prognosis of elderly patients with CHF.

In this study, we found that ALB, Hb and GNRI levels were significantly lower in the poor prognosis group compared to the good prognosis group. This result is consistent with previous studies, suggesting that malnutrition may play an important role in the poor prognosis of elderly CHF patients. Malnutrition can lead to muscle atrophy, decreased immune function and impaired organ function, which in turn affects the recovery and maintenance of cardiac function and increases re-hospitalization and mortality (7, 27). Furthermore, ALB, Hb and GNRI levels declined progressively with higher NYHA class and showed significant negative correlations with cardiac function grade. This suggests that the worse the cardiac function, the worse the nutritional status of the patients, suggesting that deteriorating nutritional status may exacerbate cardiac impairment. The possible mechanism is that ALB is a plasma protein synthesized by the liver with important functions such as maintaining plasma colloid osmotic pressure and transporting substances (28). Patients with severe cardiac insufficiency are often accompanied by gastrointestinal stasis, leading to digestive and absorption dysfunction, which in turn causes insufficient nutrient intake and malabsorption (29, 30). Meanwhile, heart failure is characterized by low-grade inflammation, a common feature of chronic disease progression, leading to the destruction of body composition, making serum ALB levels lower and further exacerbating malnutrition (30, 31). Reduced levels of Hb are more common in elderly patients with CHF, and anemia can lead to an increase in the heart's compensatory workload, exacerbating the heart's burden, as well as affecting the myocardium's oxygen supply and promote myocardial remodeling, leading to worsening heart failure (18, 32). In addition, GNRI integrates information on body weight and ALB, provides a more comprehensive nutritional assessment; its decline may indicate loss of muscle mass and protein imbalance, common in elderly CHF patients (19, 33, 34).

ROC analysis revealed that ALB, Hb and GNRI had high predictive values for poor prognosis in elderly CHF patients, with AUCs of 0.845, 0.884 and 0.896, respectively. Among them, the predictive value of GNRI was the highest, which may be related to its combined consideration of serum albumin level and body weight (35). As a simple, non-invasive nutritional index requiring no additional tests, GNRI holds practical utility in clinical practice (36). Kaplan–Meier survival analysis showed that patients with low ALB, Hb, or GNRI had significantly poorer outcomes, further underscoring the close link between nutrition and prognosis (34). Cox regression analysis showed that ALB, Hb and GNRI were independent influences on the prognosis of elderly CHF patients, which implies that in addition to the traditional clinical indexes such as LVEF, NT-proBNP and NYHA cardiac function classification, nutritional status indexes are also important in the assessment of patient prognosis. grading, indicators of nutritional status should also be taken into consideration (34, 37).

This study offers new insights for the clinical management of elderly CHF patients. First, in clinical practice, attention should be paid to assessing the nutritional status of elderly patients with CHF, and ALB and Hb levels should be tested and GNRI should be calculated regularly in order to detect malnourished patients in a timely manner. Second, for malnourished patients, individualized nutritional support programs, including dietary guidance and necessary nutritional supplementation, should be developed to improve the nutritional status and prognosis of patients. In addition, nutritional status indicators can be used as an important supplement to the prognostic assessment of elderly CHF patients, which can help clinicians more accurately predict the prognosis of patients and formulate more reasonable treatment strategies.

This study has several limitations that should be acknowledged. First, it was a single-center retrospective study with a relatively small sample size, which may introduce selection bias and limit the generalizability of our findings. Second, the results have not been validated in an independent external cohort, which is necessary to confirm their reproducibility and clinical applicability. Third, although ROC analysis was used to evaluate the predictive performance of individual nutritional indices, this study did not develop a combined predictive model. Future studies constructing prognostic models could consider incorporating more comprehensive performance metrics such as the Matthews correlation coefficient (MCC) or F1 score to provide a more robust evaluation of model classification ability. Fourth, this study included predominantly HFmrEF and HFpEF patients, with very few HFrEF cases (*n* = 3). Consequently, we were unable to conduct meaningful stratified analyses across all three HF subtypes based on LVEF classification. Future multi-center studies with balanced representation of HFrEF, HFmrEF, and HFpEF patients are warranted to clarify potential differences in nutritional status and its prognostic implications among distinct heart failure phenotypes. Fifth, this study did not systematically collect data on patients’ socioeconomic status (e.g., education, income, social support) or psychiatric comorbidities (e.g., depression, anxiety), which are known to influence nutritional intake, self-care behaviors, and clinical outcomes in heart failure. The absence of these variables limits our ability to fully elucidate the multifactorial etiology of malnutrition in this population. Sixth, the follow-up period was limited to one year; a longer observation window (e.g., two years or more) would allow for a more comprehensive assessment of rehospitalization patterns and long-term prognostic determinants. Finally, due to the retrospective design, a prospective sample size calculation was not performed. Although a *post-hoc* power analysis suggested adequate statistical power with the current cohort, the absence of *a priori* sample size estimation may affect the robustness and reproducibility of the results. Future studies should expand the sample size and adopt a multicenter prospective design to validate our findings and further elucidate the specific mechanisms through which malnutrition influences prognosis in elderly CHF patients. Additionally, future prospective studies should incorporate these psychosocial and socioeconomic dimensions, along with extended follow-up, to provide a more holistic understanding of the determinants and consequences of malnutrition in elderly patients with CHF.

In conclusion, this study confirms that nutritional status indexes (ALB, Hb, GNRI) are closely related to the prognosis of elderly patients with CHF and has a good predictive value of poor prognosis for patients. Clinicians should pay attention to the assessment of nutritional status in elderly CHF patients and incorporate it into the prognostic assessment system in order to develop a more optimal treatment plan and improve the clinical outcomes of patients.

## Data Availability

The original contributions presented in the study are included in the article/Supplementary Material, further inquiries can be directed to the corresponding author.
